# Prevalence of Cattle Trypanosomosis and Temporal Vector Distribution in Jima Arjo District, Upper Didessa Valley, Western Ethiopia

**DOI:** 10.1155/2022/2923446

**Published:** 2022-08-27

**Authors:** Debela Abdeta, Takele Deresa, Geremew Haile

**Affiliations:** ^1^Addis Ababa University, College of Veterinary Medicine and Agriculture, Bishoftu, Ethiopia; ^2^Jimma Arjo District Livestock and Fishery Office, Jimma Arjo, Ethiopia

## Abstract

Trypanosomosis is a protozoan disease, mostly transmitted by the tsetse fly, *Glossina species*, which causes severe disease of livestock in Ethiopia. The disease is also widespread across the globe especially in sub-Saharan African states. A cross-sectional study with the objectives of determining the prevalence of bovine trypanosomosis and assess the apparent densities of the disease vectors was conducted from October 2019 to July 2020 G.C in Jima Arjo district, east Wollega zone, Oromia regional state, Ethiopia. A parasitological study using conventional buffy coat technique was employed for the determination of prevalence of trypanosomosis and species was identified by Giemsa stain technique, while baited traps were used for the vector survey. The results of parasitological study revealed that the overall prevalence was 36 (8.2%) at 95% CI. From the total trypanosome positive animals 22 (5.0%), 8 (1.82%) and 6 (1.36%) of them harbor *T. congolense*, *T. vivax*, and *T. brucei*, respectively. Relatively higher prevalence (10.91%) was seen in animals with poor body condition than those with medium (7.38%) and good (5.55%), body condition though it is not statistically significant (*P* > 0.05). Higher infection rate was observed in male 26 (12.26%) than female 10 (4.39%) due to male cattle more exposed to the tsetse fly area or early released from home for drought reason. Out of the total positive animals, only 12 of them were anemic on buffy coat test of sampled blood. This justifies that animal could be positive for trypanosomosis without showing clinical sign of anemia which is the dominant sign in this disease. A total of 2185 vectors trypanosomes were collected among which 1,569 were tsetse flies and 616 were other biting flies. The density of *Glossina species* was 15.1 fly/trap/day. *Glossina morsitans submorsitans* and *G. tachnoides* were the two dominant species of tsetse flies recorded from the area. The present study indicated that tsetse and non-tsetse fly-borne trypanosomosis is a leading bottle neck for production and health of animals in Jima Arjo districts and similar case was found throughout the country which necessitates a coordinated vector and parasite control in order to alleviate the problem of the disease.

## 1. Introduction

Bovine trypanosomosis causes about 3 million deaths every year, and approximately 35 million doses of trypanocidal drugs are being administered every year to enable livestock to survive in tsetse fly-infested areas [1]. While the economic losses in cattle production alone is up to US$1.2 billion, the indirect impact engendered by the disease on the agriculture-livestock production is estimated to be about US$4.5 billion a year [1].

Livestock are the mainstay of the vast majority of African people serving as “living bank” or “living account” for rural and urban poor farmers. They contribute a large proportion of the continents GDP and constitute a major source of foreign currency earning for a number of countries. Livestock production, indeed, contributes to improve food security and poverty alleviation in developing world. Ethiopia has huge and diverse livestock population that plays an important role in the economies and livelihoods of farmers and pastoralists [2].

Ethiopia's economy is largely dependent on agriculture and livestock production. Besides its direct contribution in terms of GDP and foreign exchange, livestock provides virtually all the draught power for cultivation and transportation of agricultural crops and people in rural parts of this country [3]. African animal trypanosomosis (AAT) caused by six trypanosome species is a major livestock problem over a large tsetse fly belt areas of Africa. In sub-Saharan Africa, it jeopardizes the lives of 55 million people. The diseases are chronically debilitating protozoan disease of livestock, which is of great economic importance. The vascular trypanosomes, namely, T*. congolense* and *T. vivax*, are the most pathogenic, economically very important, and widely distributed in Ethiopia [4].

Trypanosomes transmitted by tsetse fly continue to be the major constraints of livestock production. It is also the major risk to infection in humans. In domestic animals, it greatly affects social, economic, and agricultural development of communities in tsetse flies-infested areas [5]. Resistance to conventional antitrypanosomal drugs, increasing resistance of vectors to insecticides, lack of effective vaccines, and adverse effects of the existing antitrypanosomal drugs are challenges to control of the diseases. In Ethiopia, different scholars [6–9] and have reported the use of medicinal plant and animal species for treatment and control of trypanosomiasis and as tsetse fly repellant though the disease is posing great economic and health challenges.

Eighty-eight percent of the human population and 70% of livestock exist in the highland of Ethiopia. Around 36.3% of this highland area is heavily degraded and unable to provide sufficient food for the people living on it. On the other hand, low lands of the country that accounts about 63.7% of the total land support only 12% of humans and 33% of livestock population. Substantial proportion of the fertile agricultural low lands is rendered inaccessible by the threat of tsetse fly and trypanosomosis. The northwest region is affected by tsetse and non-tsetse fly transmitted trypanosomes and [10–12]. According to Langridge [13], the tsetse fly vectors in Ethiopia are limited to the western and southern regions between longitude 33° and 38° E and latitude 5° and 12° N infesting areas which together amount to 97, 855 km^2^.

Tsetse flies found in many places of Ethiopia are fixed to specific place, and animal interface is constantly moving. Consequently, new areas being invaded and settled communities are being continually evicted by the advancing tsetse flies. Such hot spots are found in the Omo-Ghibe River Basin and its tributaries, the upper Didessa Valley, and the north and northeastern edge of Lake Abaya in Rift Valley [14]. The distribution of bovine trypanosomosis is found to be widespread covering most parts of the western and southwestern parts of the country. This area is long known to be the major tsetse fly and trypanosomosis belt in Ethiopia [15, 16]. The area is one of the wettest and agriculturally productive parts of the country. Estimates made decades ago reported that 180,000–220,000 km^2^ land in the western and southwestern parts of the country to be suitable for tsetse fly, the biological vector of trypanosomosis. A recent estimate made by Leta et al. [17] reported that 140,000 km^2^ of fertile agricultural land which is roughly 12% of the country's landmass is found to be a suitable habitat for tsetse fly. In line to this estimates, different fly per trap day (1.15 in east Wollega, 2.42-11.9 in Halu district, 11.9 in Hawa Galan, and 4.3 in Lalo Kile) as reported by [18–21], respectively, indicating vectors are distributed throughout the country. Most data in Ethiopia and elsewhere indicated that the female tsetse fly have longer life span than males [19]. Increasingly tsetse fly and trypanosomosis control schemes become concentrated in selected areas of high priority; the areas where control is technically feasible and where economic returns are considerable [22]. In an attempt to solve this bottleneck problem of the country, it necessitates further updated research on description of host, agent, and environment relationship in Jima Arjo. The present research was initiated to assist in decision making and hence administer appropriate tsetse fly and trypanosomosis control. Therefore, the present study aims at determining the prevalence of cattle trypanosomosis and to measure the type, apparent density of tsetse flies, and other mechanical vectors in selected peasant associations (PAs) of Jima Arjo district.

## 2. Study Materials and Methods

### 2.1. Study Area Description

The study was conducted in Jima Arjo district, east Wollega administrative zone of Oromia regional state western part of Ethiopia bordering to Didessa River Valley Basin from October 2019 to July 2020 G.C. The district covers an area of 75,553 hectares and bordered by Nunu Qumba district in east, Dabo Hana district in west, Leka Dulecha district in north, and Bedelle district in south. The climatologic condition alternates with long summer rain fall (June to December) and winter dry season (January to May) with mean annual rain fall of 824-2616 mill meters, and the annual mean temperature for most part of the district is 15.7-24°C, and the elevation varies from 1200-2500 meter above sea level (m.a.s.1). The vegetation type of the area is characterized by common riverine vegetation. The area has been a virgin land and with reserved vegetation. It is rich with wild game animals in many river systems and savanna. Some of these wild animals include baboons, monkey, African buffalo, lion, bush pig, crocodile, hyena, snakes, and others (livestock development and fisher resource office of Jima Arjo district). The livestock populations that are found in Jima Arjo district include cattle 112,101, sheep 30,546, goats 20,821, horses 1115, mule 315, donkey 10,424, and poultry 63,902 [23]. Among these animals, cattle are the dominant species raised in the area.

### 2.2. Study Population

The study was conducted on indigenous cattle breed of all age groups of both sexes in selected PAs which were managed under extensive husbandry system in communal herding. The age estimate was based on owner's information and checking the removal of incisor teeth after one year. Accordingly, cattle either male or female age less than 2 years were classified as calves, those above 2-5 years were classified as young, and those >5 years were adult [24]. The body condition of animals were recorded by classifying animals into three groups as good, medium, and poor based on the appearance of ribs and dorsal spines [25]. The altitude of the study area was low land and high land in four purposively included PAs.

### 2.3. Study Design

A cross-sectional study was conducted from October 2019 to July 2020 to determine the prevalence of cattle trypanosomosis and the distribution and density of tsetse fly and other biting flies in four purposively selected PAs (Hara, Abote Didesa, Hunde Gudina, and Meta) in Jima Arjo district, Upper Didessa River Valley.

### 2.4. Sampling Technique and Sample Size Determination

A simple random sampling technique was followed to select the study animals. The sample size was determined based on previously conducted study by [26] at Haro Tatesa area Bedelle district of 32.25% expected prevalence and absolute desired precision of 5% at 95% confidence level. The desired sample size was calculated using the formula given by Thrusfield [27].

(1) $$\begin{array}{c} n=\frac{1.962\times \text{pexp}\left(1-\text{pexp}\right)}{d^{2}}, \end{array}$$

where *n* is the required sample size, Pexp is the expected prevalence, and *d* is the required precision.

Accordingly, the calculated sample size required was 336; however, to increase the precision, approximately one-third of the sample size was added; hence, 440 heads of cattle were included in the study area.

### 2.5. Sample Collection and Laboratory Analysis

#### 2.5.1. Parasitological Survey

Blood samples were collected in to heparinized capillary tubes after piercing the ear vein using the lancet. One end of the capillary tube was sealed with sealant and centrifuged at 12,000 rpm for five minutes to separate the blood cells and to concentrate trypanosomes using centrifugal force as buffy coat. Then, PCV was read using hematocrit reader. Animals was considered anemic, if the PCV<24 and nonanemic if above this value [28]. The capillary tubes were then broken just below buffy coat and expressed on microscope slide and mixed and covered with a 22 × 22 mm cover slip. It was then observed under ×40 objective of microscope using dark ground buffy coat technique to detect the presence of parasites, and for positive samples, Giemsa stain of thin blood films was made [29, 30].

*(1) Entomological Survey*. For the entomological study, tsetse flies and other biting flies were collected by monoconical traps deployed in different positions of the study areas of different PAs. A total of 52 monoconical traps were deployed in the river side and savanna area at approximately 100 m-200 m and apart for three days. Twenty-six monoconical traps were deployed during wet season, and 26 monoconical traps were deployed during dry season. In all the traps, acetone, octanol, and fenol (cow urine) were used as bait to attract the flies. All odors were placed on the ground about 30 cm upwind of the trap [31]. Fly catch per trap per day (f/t/d) was determined to calculate the fly density and distribution [32]. Species of the caught flies were identified based on the morphology and characteristics such as size, color, proboscis, and wing venation structures at genus level [33]. Sexing was also done for the flies just by observing the posterior end of the ventral aspect of abdomen by hand lens; as a result, male flies are easily identified by enlarged hypophgeum.

### 2.6. Data Management and Analysis

For the analysis, data was collected and entered in to MS Excel sheets and analyzed using SPSS versions 20. Differences between disease status and different risk factors were assessed using Chi-square test. Statistical significance differences among parameters were tested at probability levels of *P* < 0.05 and 95% CI. Finally, the fly population is calculated by dividing the number of flies caught by the number of traps deployed and number of days of deployment and expressed as fly/trap/day.

## 3. Results

From 440 animals examined, 36 were found positive for bovine trypanosomosis with overall prevalence of 8.2% in the study area. This prevalence was determined to be 10.1% in Meta, 9.09% in Abote Didesa, and 7.27% in Hunde Gudina.  Table 1 summarizes the prevalence of bovine trypanosomiasis and corresponding infection rate in four selected PAs.

Out of the trypanosomosis positive cattle, 5.55%, 7.38%, and 10.91% were recorded in good body condition, medium, and poor body condition animals, respectively. A relatively higher prevalence rate 10.91% was seen in animals with poor body condition than that of those with good and medium body condition animals. From different age groups included in this study, 3.66%, 8.82%, and 9.74% prevalence rate were recorded in <2, 2-5, and>5 years of age, respectively. A comparison of trypanosome infection between two sexes indicated that male and female were made. The overall prevalence in male and female was 12.26% and 4.29%, respectively. The prevalence of trypanosome in male was higher than female with (*χ*^2^ = 2.4064; *P* > 0.05) though it was not statistically significant.  Table 2 summarizes the association between the prevalence of disease and different risk factors in selected PAs.

*Trypanosoma congolense*, *T. vivax*, and *T. brucei* were the trypanosome species identified by Giemsa-stained thin blood smear examination. Among the total 36 trypanosome infections detected in blood of tested animals, 22 (61.1%), 8 (22.2%), and 6(16.67%) were due to T*. Congolense*, *T. vivax*, and *T. brucei*, respectively (Table 3).

### 3.1. Hematological Survey

The PCV of individual animal was measured for the assessment of degree of anemia. Out of the total examined animals, 36 (8.2%) of them were positive, and only 12 (33.3%) of anemic animals were found to be positive for trypanosomosis. From 404 trypanosome negative animals, 110 (27.2%) were under anemic category (PCV<24%). From the obtained result mean PCV (%) values, there was no statistically significant difference (*P* > 0.05) between infected and noninfected animals (Table 4).

### 3.2. Entomological Survey

A total of 52 monoconical traps deployed approximately 100-200 m apart and left in position for three days each for dry and wet season (three days for wet seasons and three days for dry seasons). From all traps deployed, 2,185 flies were caught. The flies belong to *Glossina m. submoristans*, *G. tachionoides*, *Stomoxys*, *Tabanus*, and *Heamatopota* (Table 5).

The entomological monitoring showed that 15.1 f/t/d, 3.2 f/t/d, 0.8 f/t/d, and 2.0 f/t/d for *Glossina* species, *Stomoxys*, *Tabanus*, and *Heamatopota*, respectively, with the overall apparent fly density of 21.0 f/t/d. flies caught per trap were high during wet season than dry season (Table 6).

Five hundred thirty seven (537) (34.23%) of male and 1,032 (65.77%) of female *Glossina* species were caught during the study period (Table 7). The presence of *Glosinna* species and different season is statistically associated (*P* = 0.03).

## 4. Discussions

Overall prevalence 8.2% of trypanosomosis was recorded during the study period. This finding was close with reports of Mekuria and Gadisa (2011) who reported 12.41% in Metekel and Awi zones and Shimelis et al. who reported 12.0% in Dembecha and Jabitehenan area. The current finding was lower than 14.2% of Abraham and Tesfaheywot Arbaminch araea and 17.33% of Zeleke report from South Ethiopia [34–37]. Moreover, Efa [38] reported lower (4.39%) prevalence rate from the same district. The lower prevalence of cattle trypanosomes in the present study might be due to the presence of strategic tsetse fly control measures carried out by the National Tsetse and Trypanosomosis Investigation and Control Center and Bedelle Regional Veterinary Laboratory in the study area for the last years. Though most of the Trypanosome species are developing resistance against existing drugs in addition to the toxicity of the drugs, farmers are still using available chemotherapeutic and chemoprophylactic drugs.

*Trypanosoma congolense* was the predominant species of trypanosome followed by *T. vivax* and *T. brucei* in the study area. This finding is in agreement with the finding reported by Abiy [39] in Goro district of the south Ethiopia and Cherenet et al. [40] in Amhara region and Efa [38] in different PAs of Jimma Arjo district. The predominance of *T. congolense* infection in cattle suggests that the major cyclical vectors or *Glossina* species (*G.m. submorsitans*, *G. tachinoides*, and other*s*) are more efficient transmitters of *T. congolense* than *T. vivax* [41]. The higher occurrence of *T. vivax* most likely indicates the local transmission in the non-tsetse-infested area by biting flies. It is indicated that *T. vivax* can adapt to a non-tsetse fly-dependent transmission cycle [42].

The prevalence of trypanosomes was relatively higher in male than female cattle with significant difference between the sex groups (*P* < 0.05). This finding is in agreement with Tewelde [43] in western Ethiopia and Efa [38] at Jimma Arjo district. This finding is not in agreement with study done by [44–46] who reported different results from Hawa Galan, Wolaita zone Kindo Konish district, Guto Gida district of east Wollega zone, and Mandura district northwest Ethiopia, respectively. The high prevalence in males may be related stress by plough and early release from home for this purpose though both male and female are equally affected in high tsetse fly challenge area.

The rate of infection in cattle increased with age. This finding is in agreement to report of [47, 48] in Ghibe Valley and Debre Elias district, northwestern, Ethiopia, respectively, which indicated that suckling calves do not go out with their dams but graze at homesteads until they are weaned. It was also suggested that calves are slightly protected by maternal antibodies [49]. This could be associated to the fact that young and adult animals travel long distance for grazing and draught as well as harvesting crops in areas of high tsetse fly challenge than calves.

The finding showed that the rates of infection in poor body condition animals were higher than that of medium and good body condition animals. This result agreed with previous reports by and [38, 49–51]. These may be due to reduced performance of animals created by lack of essential nutrients and poor management by the animal owner. In contrast, trypanosomosis is a chronic disease as stated by [52]. This indicated that chronically infected animals often die secondary to poor body condition, immunosuppression, and concurrent infections.

Anemia was one the leading clinical sign of trypanosomosis. This finding was in agreement with previous reports [36, 46] in Hawa Gelan district, west Wollega zone, western Ethiopia, and in Arbaminich, respectively. However, in large number of animals, 110 (27.7%) had anemia without having trypanosomosis infection. This suggests that even though anemia is a characteristic of trypanosomosis; other diseases, such as fasciolosis, gastrointestinal parasitism, vector borne disease, and nutritional deficiencies, can cause reduced PCV profile of animals [28].

Higher prevalence rate the disease was observed in Meta and Abote Didesa PAs. This is due to difference in altitude in which these PAs are close to Didessa Valley. Animals in both high land and low land are infected by the disease indicating the wide distribution of tsetse flies and other mechanical vectors across different agroecological zones as reported by Ayele et al. [2]. Different agroecological zone also contributed for difference in tsetse and disease distributions.

The entomological survey indicated apparent tsetse fly density of 5.03, 10.04, 3.2, 0.8, and 2.0 fly/trap/day for *G. m. submorsitans G. tachinoides*, *Stomoxys*, *Tabanus*, and *Heamatopota*, respectively. The overall mean catch of tsetse flies was 21.0 flies/trap/day. This finding agreed with [53] who reported 16 to 22.4 flies/trap/day in Tana Beles Valley and higher than [54] who reported mean catchment of 10.68 flies/trap/day in upper Didessa Valley. The population of *G. tachinoides* was higher than *G. m. submorsitans*. This could be related to varieties in the type of vegetation and riverine. Female tsetse fly accounts for 65.77% catch during this study. This result was slightly in agreement with the work of Bancha [55] and Bekele [56] who reported 60 and 63.2 catchment of female, respectively. Female *G. tachinoides* showed relative high density than others in which this result is found to be highly consistent with the previous study by Bekele et al. [57] in which they reported 63.2% female *G. tachinoides* [58] associated higher catches of female *G. tachinoides* to be attributable due to their longer life span (average of 8 weeks) than males living about 4 weeks, so that more catch of females could appear.

## 5. Conclusion and Recommendations

The current study trypanosomosis poses major constraint of animal health and production in the study area. *Trypanosoma congolense*, *T. vivax*, and *T. brucei* were responsible for cattle trypanosomosis with anemia as a cardinal sign. Tsetse and other biting flies are associated with the disease. In both dry and wet seasons, large numbers of tsetse flies were caught, and the severity of the problem was evident. It is indicated that both sexes and all age groups included in this study were found infected though the rate is different. It is necessary to design and implement strategies for controlling trypanosomosis, including vector control and chemotherapy.

## Figures and Tables

**Table 1:** The prevalence of bovine trypanosomiasis in the study PAs.

Name of PA	Number of animals	Positive	Prevalence (%)	*χ* ^2^	*P* value
	Examined				
Hara	110	7	6.36		
A/Didesa	110	10	9.09		
H/Gudina	110	8	7.27		
Meta	110	11	10.0	7.35	0.53
*Total*	*440*	*36*	*8.2*		

*χ* ^2^ = Chi-square test.

**Table 2:** Association between prevalence and risk factors.

Variables	Variables classification	Noninfected	Infected	Prevalence (%)	*χ* ^2^	*P* value
BCS	Good	126	7	5.55	1.35	0.510
	Medium	149	11	7.38		
	Poor	165	18	10.91		
Age	<2years	82	3	3.66	3.38	0.781
	2-5years	204	18	8.82		
	>5years	154	15	9.74		
Sex	Male	212	26	12.26	18.64	0.046
	Female	228	10	4.39		
Altitude	High L	100	8	8.00	.006	1.0
	Low L	340	28	8.23		

*χ* ^2^: Chi-square.

**Table 3:** Prevalence of identified trypanosomes species in the study area.

Name of PAs	No. of examined	Species of trypanosome found in number			Prevalence	*χ*2	*P* value
		*T. congolense*	*T. vivax*	*T. brucei*			
Hara	110	4	1	2	7		
Meta	110	6	3	2	11		
A/Didesa	110	7	2	1	10	6.89	0.17
H/Gudina	110	5	2	1	8		
*Total*	*440*	*22*	*8*	*6*	*36*		

T: trypanosome; H/Gudina: Hunde Gudina; A/Didesa: Abote Didesa.

**Table 4:** Comparison of mean PCV value between parasitemic and aparasitemic cattle.

Condition	Number examined	PCV<24%	PCV>24%	*χ*2	*P* value	
Parasitemic	36	12 (33.3%)	24 (66.7%)	0.62	0.44	
Aparasitemic	404	110 (27.2%)	294 (72.8%)			
*Total*	*440*	*122 (27.7%)*	*318 (72.3%)*			

**Table 5:** The apparent density of fly at different PAs in study area.

Pas	No. of traps deployed	Fly species				Total	F/T/D
		*Glossina*	*Stomoxys*	*Tabanus*	*Heamatopota*		
Hara	12	293	26	18	30	367	15.3
A/Didesa	14	607	155	15	54	831	29.7
H/Gudina	12	415	112	23	80	630	26.3
Meta	14	254	35	23	45	357	12.8
*Total*	*52*	*1569 (15.1)*	*328 (3.2)*	*79 (0.8)*	*209 (2.0)*	*2185*	*21.0*

F/T/D: fly per trap per day; A/Didesa: Abote Didesa; H/Gudina: Hunde Gudina.

**Table 6:** The overall apparent density of fly during different seasons in the study area.

Season	Traps deployed	Fly species				Total	F/T/D
		*Glossina*	*Stomoxys*	*Tabanus*	*Heamatopota*		
Wet	26	1112	258	39	128	1537	29.6
Dry	26	457	70	40	81	648	12.5
*Total*	*52*	*1569* *15.1*	*328* *3.2*	*79* *0.8*	*209* *2.0*	*2185*	*21.0*

**Table 7:** The seasonal apparent density of *Glossina* species in study area.

Season	Glossina species				Total	F/T/D	*χ*2	*P* value
	*G. moristans*		*G. tachinoides*					
	M	F	M	F				
Wet	98	265	284	465	1112	21.4	18.96	0.03
Dry	48	113	107	189	457	8.8		
*Total*	*146*	*378*	*391*	*654*	*1569*	*15.1*		

## Data Availability

The author will provide all data up on request.
